# L’apport de la dermoscopie dans le carcinome basosquameux

**DOI:** 10.11604/pamj.2016.25.252.11345

**Published:** 2016-12-21

**Authors:** Hakima Elmahi, Asmae Lahlou, Hanane Baybay, Salim Gallouj, Fatima Zahra Mernissi, Fatima Zahra Reggad, Taoufik Harmouch

**Affiliations:** 1 Département de Dermatologie, CHU Hassan II, Fès, Maroc; 2 Département d’Anatomo-pathologie, CHU Hassan II, Fès, Maroc

**Keywords:** Carcinome basosquameux, carcinome spinocellulaire, carcinome basocellulaire, dermoscopie, Basosquamous carcinoma, squamous cell carcinoma, basal cell carcinoma, dermoscopy

## Abstract

Le carcinome basosquameux (CBS) est un cancer cutané rare qui présente des zones de carcinome basocellulaire (CBC) et de carcinome épidermoïde (SCC) et une zone de transition entre elles. Cependant, les caractéristiques dermoscopiques du BSC ne sont pas bien décrites dans la littérature, sauf deux études. Le but du présent cas était de mieux identifier et clarifier la contribution de la dermoscopie dans le diagnostic du BSC, malgré que la confirmation reste toujours histologique.

## Abstract (en)

Basosquamous carcinoma (BSC) is a rare skin cancer which has areas of basal cell carcinoma (BCC) and squamous cell carcinoma (SCC) and a transition zone between them. However, dermoscopic features of BSC are not well described in the literature, except for two studies. The aim of this case study was to better identify and clarify the role of dermoscopy in the diagnosis of BSC, although histological confirmation is necessary.

## Introduction

Le carcinome basosquameux (CBS) est une tumeur rare, potentiellement agressive, caractérisée par les caractéristiques cliniques et pathologiques du carcinome basocellulaire (CBC) et du carcinome épidermoïde (CSC) [1]. Bien que les caractéristiques dermoscopiques du CBC et du CSC aient fait l´objet d´études approfondies, Celles de CBS restent inconnues [1]. Nous rapportons un cas CBS dont le diagnostic a été redressé par la dermoscopie, bien que la confirmation soit toujours histologique.

## Patient et observation

**Figure 1:**
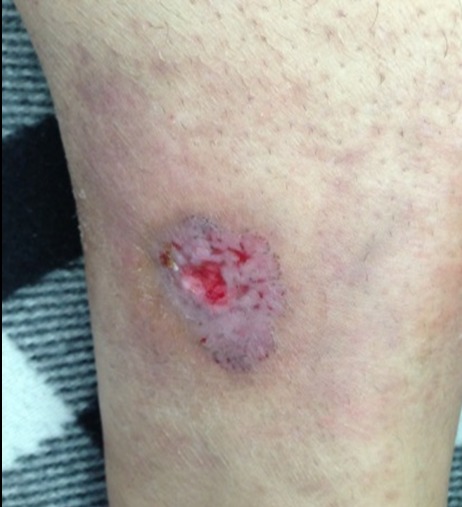
Image clinique: ulceration au niveau de la jambe

**Figure 2:**
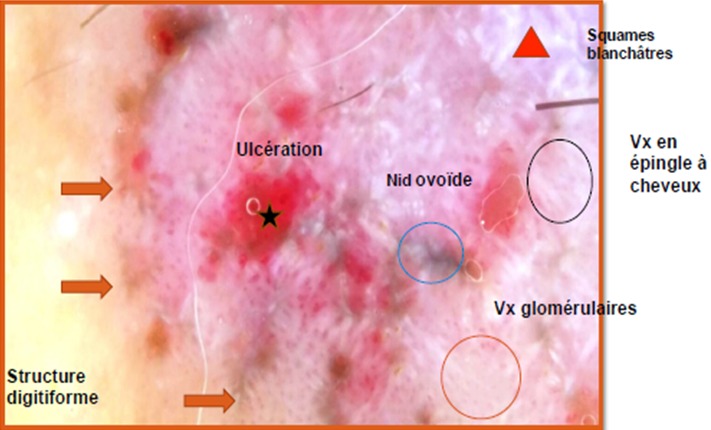
Image dermoscopique

**Figure 3:**
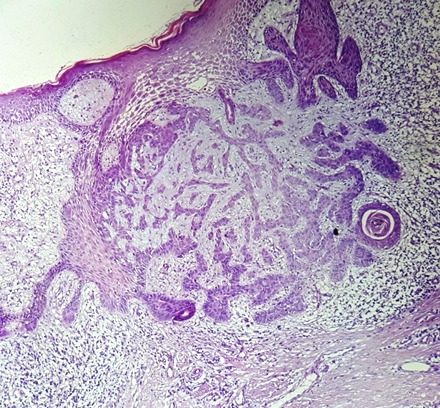
Coupe histologique du carcinome basocellulaire

**Figure 4:**
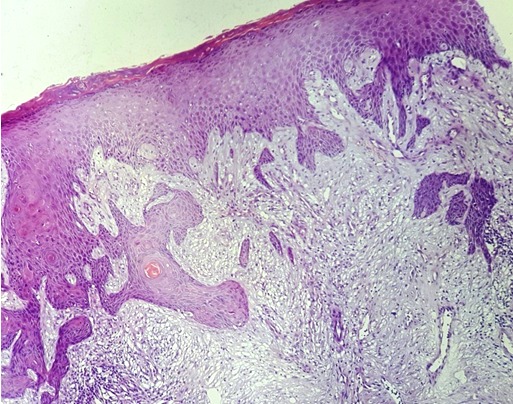
Coupe histologique du carcinome spinocellulaire

Homme de 59 ans, diabétique non insulinodépendant et hypertendu, hospitalisait pour prise en charge d'un carcinome spinocellulaire (CSC) confirmé histologiquement après une biopsie d'une Lésion de la jambe gauche, indolore, légèrement prurigineuse, évoluait depuis 10 mois. L'examen clinique trouvait une ulcération de 3 cm, bien limité surface propre recouverte de squames blanchâtres par endroit reposant sur une peau légèrement atrophique (Figure 1). La dermoscopie a confirmé le diagnostic du carcinome spinocellulaire en objectivant une vascularisation glomérulaire et en épingles à cheveux entourée d'un halo blanchâtre avec des squames (Figure 2). D'autres lésions ont été retrouvées, notamment : des nids ovoïdes, une ulcération au centre et des structures digitiformes évoquant le diagnostic d'un carcinome basocellulaire associé (Figure 2). Le diagnostic d'un carcinome plutôt mixte (Figure 3, Figure 4) a été évoqué et confirmé pat l'histologie d'une biopsie-exérèse avec marge de 10 mm. Le bilan d'extension était négatif.

## Discussion

Le carcinome mixte (CM) ou basosquameux (CBS) ou composite est une entité rare, potentiellement agressive, ayant les caractères cliniques et pathologiques des CBC et CSC, considérait comme une variante du CBC, mais actuellement se définit comme un complexe tumoral [1]. Sa clinique est non spécifique, il peut mimer aussi bien des tumeurs malignes que bénignes [1]. Ceci entrave sa reconnaissance et conduit à une gestion inappropriée avec des conséquences indésirables. Bien que ses caractères dermoscopiques restent peu étudiés [1, 2], elles regroupent celles du CBC (nids ovoïdes, ulcération, croutes de sang, vaisseaux arborisants et des structures pigmentées : roue dentée, structures digitiformes..) et de CSC (vascularisation glomérulaires, en épingle à cheveux et linéaires irrégulières, kératine, zone blanchâtre sans structure, squames blanchâtres) [1, 2]. Le diagnostic dermoscopique du carcinome mixte nécessite la présence au moins une caractéristique à la fois CSC et BCC [1, 2]. La confirmation reste toujours histologique. Cette entité est potentiellement agressive avec un potentiel de récidive locale et métastase ganglionnaire et à distance ; d'où l'intérêt d'un diagnostic précoce et de l'exérèse obligatoire afin d'éviter la morbidité significative et même la mortalité liée à ce carcinome agressif de la peau. Plusieurs cas cliniques et des études de cas ont rapporté l'utilité de la dermoscopie afin de poser le bon diagnostic [3, 4]. La microscopie confocale par réflectance in vivo peut constituer également une aide au diagnostic dans ces lésions d'interprétation difficile [5]. Le bilan, le traitement et le pronostic sont similaire à celui du CSC.

## Conclusion

En concluant, le diagnostic dermoscopique du CBS est retenu par la présence au moins une caractéristique à la fois du CSC et du CBC [**1**, **2**]. L'exactitude de ces critères proposés pour différencier CBS d'autres tumeurs malignes et bénignes de la peau nécessite une évaluation par d'autres études.
